# Iron Deficiency Anemia: Focus on Infectious Diseases in Lesser Developed Countries

**DOI:** 10.1155/2011/260380

**Published:** 2011-05-15

**Authors:** Julia G. Shaw, Jennifer F. Friedman

**Affiliations:** ^1^Public Health Program, Division of Biology and Medicine, Brown University, Providence, RI 02912, USA; ^2^Lifespan Hospital Center for International Health Research, 55 Claverick Street, Suite 100, Providence, RI 02903, USA; ^3^Warren Alpert School of Medicine, Brown University, Providence, RI 02912, USA

## Abstract

Iron deficiency anemia is thought to affect the health of more than one billion people worldwide, with the greatest burden of disease experienced in lesser developed countries, particularly women of reproductive age and children. This greater disease burden is due to both nutritional and infectious etiologies. Individuals in lesser developed countries have diets that are much lower in iron, less access to multivitamins for young children and pregnant women, and increased rates of fertility which increase demands for iron through the life course. Infectious diseases, particularly parasitic diseases, also lead to both extracorporeal iron loss and anemia of inflammation, which decreases bioavailability of iron to host tissues. This paper will address the unique etiologies and consequences of both iron deficiency anemia and the alterations in iron absorption and distribution seen in the context of anemia of inflammation. Implications for diagnosis and treatment in this unique context will also be discussed.

## 1. Introduction

Anemia is a worldwide public health problem, with global prevalence estimated at 24.8% (95% CI: 22.9–26.7). The majority of the global disease burden of anemia is shouldered by the developing world, with prevalence in Africa and South East Asia as high as two thirds among children under five, and nearly half among women [1]. The World Health Organization (WHO) has classified anemia as a severe public health problem (prevalence ≥ 40%) for children under five in 69 countries, and for pregnant women in 68 countries [1]. The causes of anemia in the developing world are multifactorial and include nutritional deficiencies, extra-corporal blood loss, higher prevalence of hemoglobinopathies, and inflammation. These etiologies often coexist and are difficult to distinguish due to limited diagnostic capabilities in resource-poor settings. High fertility rates also contribute to the burden of anemia among pregnant women and infants, with short interbirth intervals placing women at higher risk for iron deficiency anemia with concomitant increased risk of iron deficiency for the newborn [2–4]. This papaer will focus primarily on the etiology of iron deficiency anemia in the context of infectious diseases endemic in lesser developed countries.

The WHO notes that anemia is often used as an inappropriate proxy for iron deficiency, given the coexistence of many etiologies [5]. Recent studies demonstrate that the role of anemia of inflammation in the pathogenesis of anemia in the developing world is underestimated [6], as it plays a central role in the context of infectious diseases prevalent in these regions [7–14]. Though both iron deficiency anemia and anemia of inflammation ultimately lead to host tissue iron insufficiency, it is likely that health outcomes related to each differ. Improved diagnostic tools to define etiology, including multiple concurrent etiologies, are an important first step to understanding how different causes of anemia adversely impact health outcomes. Due to recent advances in our understanding of the etiology of anemia of inflammation, and specifically the role of hepcidin in altering iron metabolism, improvements in diagnostic tools to distinguish iron deficiency anemia and anemia of inflammation will be increasingly possible.

## 2. Iron Deficiency in the Developing World

Iron deficiency is thought to affect the health of more than one billion people worldwide [15]. The WHO/World Bank has ranked iron deficiency anemia as the third leading cause of Disability Adjusted Life Years (DALY's) lost for females of childbearing age [16], and among the top 10 disease burdens for men in this age group (15–44 years) [16]. Among infants, iron deficiency is the most common micronutrient deficiency and cause of anemia worldwide [17, 18]. Young children and women in lesser-developed countries (LDCs) are most significantly affected by iron deficiency anemia [15], which has numerous causes specific to the developing world including blood loss due to poor dietary iron intake, high fertility rates, and fecal iron loss in the context of parasitic infections such as hookworm, schistosomiasis, and trichuriasis. 

Iron deficiency occurs at a higher prevalence among women than among their male counterparts due to menstrual iron losses and the extreme iron demands of pregnancy (approximately two times those of a nonpregnant state). The growing fetus requires a large supply of iron, which is taken up from maternal blood via transferrin-receptor mediated endocytosis [19]. Once maternal iron stores are depleted, she becomes anemic and transfer of iron to the developing fetus may be compromised [2, 20]. In utero acquisition of iron determines newborn iron stores [21] and remains associated with iron status at 9 [22] and 24 months of age [23]. Thus, infants who are born with insufficient iron stores are unlikely to reacquire adequate iron supplies, particularly in LDCs where maternal breast milk may contain inadequate iron and weaning foods are notoriously iron poor. This risk persists, and even increases, for young children in LDCs where diets are often poor in bio-available iron, particularly in areas where iron fortification programs are not in effect. Young children remain a particularly vulnerable group due to the increased iron requirements during periods of rapid growth, which are almost 10 times higher per kilogram of body weight than that of an adult male.

One of the major consequences of iron deficiency for infant health is cognitive impairment, as infancy is a key period for brain growth and development, and iron uptake into the brain is maximal during this period [24]. Iron is required for several important brain functions including myelination of nerve fibers, energy metabolism, and acting as a cofactor for a number of enzymes involved in neurotransmitter synthesis [21, 25]. Many well-designed studies conducted in LDCs among infants and young children have demonstrated that iron supplementation leads to improvements in specific domains of cognition [26, 27]. Domains that are more amenable to iron therapy include language and motor development [26]. Of note, some studies have found that the effect of treatment is modified by baseline characteristics, such that children with poorer nutritional or hemoglobin status at baseline are more likely to benefit [28]. Further, many studies have demonstrated impairment of development even at mild or moderate degrees of iron deficiency anemia [26, 27, 29, 30]. It should be noted, however, that a number of randomized controlled trials have demonstrated no benefit of iron therapy on cognitive performance. This may be due to short periods of followup, capture of domains of cognition that may not be as sensitive to iron insufficiency, or examination of domains of cognition that may be related to iron insufficiency, but are no longer “plastic” or amenable to treatment [31]. 

Iron deficiency is also thought to cause decreased work capacity [32]. This has more significant implications for LDCs, where a greater proportion of a nation's gross national product is related to manual labor. In a study based in China, migrant schoolchildren with severe iron deficiency were found to have decreased aerobic capacity and habitual physical activity [33]. In addition, studies have been conducted examining the relationship between treatment for helminth infections and physical work capacity. Studies have demonstrated increased work capacity following treatment for both schistosomiasis and hookworm [34, 35]. Though much of this benefit is likely due to improvements in iron status with treatment, it should be noted that improved work capacity in these contexts might also be due to improved nutritional status and reduction in parasite-related symptomatology.

## 3. Infectious Diseases and Iron Deficiency

### 3.1. Hookworm

Hookworm infection is one of the most important parasitic diseases in humans in terms of Disability Adjusted Life Years (DALYs) lost, outranking schistosomiasis, African trypanosomiasis, Chagas disease, and leprosy [36, 37]. Much of the burden of hookworm is due to extra-corporeal iron loss [38], and interventions to treat hookworm infection have demonstrated significant improvements in hemoglobin [39]. Iron-deficiency anemia resulting from chronic intestinal blood loss due to hookworm infection often causes long-term morbidity [40, 41]. Blood loss is caused predominantly by parasite release of coagulases, causing ongoing blood loss in the stool, rather than actual blood consumption by the parasite. For example, *A. duodenale* is estimated to cause up to 0.25 mL of blood loss per worm per day [41]. A hookworm burden of 40–160 worms (depending on the iron status of the host) is associated with iron deficiency anemia [42]. Women of childbearing age, pregnant women, and young children are at greatest risk for hookworm-associated iron deficiency anemia due to low iron stores resulting from diets insufficient to meet demands.

The development of hookworm-related iron deficiency anemia depends on the level of an individual's iron stores, the intensity of infection, and the infecting species as *A*. *duodenale* causes a greater blood loss than *N. americanus*. A study conducted in Tanzania examined 525 school-age children, comparing the degree of anemia and iron deficiency among children infected with each species. It was found that among children with *N. americanus*, anemia was prevalent in 60.5% and iron deficiency anemia in 33.1%. Among children infected with *A. duodenale*, prevalence was 80.55% and 58.9%, respectively, suggesting that *A. duodenale* is associated with a greater burden of iron deficiency [40]. 

Hookworm is unique among helminths in that infection intensities tend to peak in adulthood [43]. Rather than peaking in childhood and adolescence and then declining as is the case of many other parasitic infections, the intensity of hookworm infection follows a steady rise during childhood and does not reach a peak or plateau until adulthood [42]. A study conducted in Hainan Province, China, which examined risk factors associated with *N. americanus* in people 50 years of age and older, found that age still accounted for 27% of the variation in the intensity of hookworm infection [44]. This is likely due to the fact that neither age- nor exposure-related immunity develops in the majority of hookworm-infected people [45]. This further supports the concern that hookworm infection may continue to affect work capacity among the most productive members of society.

The association between hookworm infection intensity and increasing age has serious implications for women of reproductive age who are already at risk for anemia due to low iron stores related to poor dietary intake, menstrual blood loss, and pregnancy. Hookworm is likely a major contributor to the adverse birth outcomes associated with iron deficiency anemia, most notably low birth weight [46]. In a randomized, double blind placebo controlled trial among pregnant women in Sri Lanka, the proportions of stillbirths and perinatal deaths were significantly lower in the group treated with mebendazole compared to the control group (1.9 versus 3.3%, 0.55 (95% CI 0.4–0.77)), as was the proportion of low-birthweight babies (1.1 versus 2.3%, 0.47 (95% CI 0.32–0.71)). Of note, the authors found a slight increase in the odds for major congenital defects among the women who received mebendazole, “contrary to medical advice,” during the first trimester only (odds ratio 1.66 (0.1–3.56), *P* = .23). In a separate study conducted in Uganda, where the prevalence of maternal anemia was 40% at enrollment, investigators found no overall effect of albendazole on maternal anemia or birthweight, but a suggestion of benefit for anemia among women with moderate to heavy intensities of hookworm infection at baseline (OR 0.45; 95% CI, 0.21–0.98; *P* = .15 for interaction). 

There has been significant debate regarding the role of soil-transmitted helminths (STH), which include hookworm, ascaris, and trichuris, in cognitive impairment. Though other mechanisms have been discussed [47], iron deficiency is consistently proposed as a primary mechanism leading to cognitive impairment. Many factors have contributed to conflicting results. Of note, early cross-sectional studies that demonstrated relationships between soil-transmitted helminths (STH) and cognitive function have been criticized for lack of control for important confounders such as SES [47]. While some cross-sectional studies that have adjusted for potential confounders have found a relationship between cognitive performance and STH infection [48], other studies have not. Further, randomized controlled trials examining the effects of treatment for STH have had differing results [28, 49]. A recent meta-analysis addressing the topic of treatment for STH and cognitive outcomes concluded that a relationship between antihelmintic treatment and improved cognitive performance could not be determined [50]. This papaer noted that the quality of even the randomized controlled trials was generally poor. This meta-analysis was highly controversial, with many ensuing published and unpublished differences of opinion [51–54]. One major critique was that baseline infection status of the children was not determined in the majority of trials included, such that both infected and uninfected children were randomized and treated. This “mass treatment” approach leads to an underestimation of the effect of treatment among those who are infected with STHs. In fact, one critique noted that among the studies that were able to stratify analyses by baseline infection status, a treatment effect was seen [49]. In addition, studies were conducted in parts of the world with varying intensities of each STH and with drugs that have varying effect on each STH. In addition, short follow-up periods in the context of randomized controlled trials may have also obscured the ability to capture improvements, as children likely need to recover iron stores before significant gains can be appreciated. Finally, children with long-standing helminth infections may develop deficits in the actual ability to learn and process new information, which may take long periods of time to develop or reacquire. Conclusions from this meta-analysis are thus limited to the impact of a mass treatment approach, which may not lead to significant improvements in cognitive performance in a target population with varying intensities of infection. It is likely, however, that among children who are infected, particarly at higher intensities of infection, treatment does lead to improvement in specific areas of cognitive function [49]. Despite these controversies, expected improvements in cognitive and school performance have been among the key reasons cited in recent initiatives for widespread, regular antihelmintic treatment of school children in endemic areas [42, 55].

## 4. Schistosomiasis: Iron Deficiency Anemia and Anemia of Inflammation

A number of cross-sectional studies have examined the relationship between the three most prevalent human schistosomes and anemia [8, 56–62]. However, due to the numerous nutritional deficiencies and infectious diseases that co-exist with schistosome infections, which are also related to increased risk for anemia, careful control for confounding variables or use of an experimental design is necessary to quantify this association. Recent cross-sectional studies have better adjusted for potential confounders, allowing for an improved estimation of the true relationship between schistosomiasis and anemia [59–61]. These recent studies have observed an inverse relationship between *S. japonicum* intensity of infection [60, 61] and hemoglobin, and *S. haematobium* infection and risk of anemia among young children and adolescents [59]. 

Many randomized controlled trials have found that therapy with praziquantel and albendazole [63–65] or metrifonate [66, 67], which is efficacious against schistosome and hookworm infections, leads to improvements in hemoglobin. The use of combination therapy makes inferences regarding the schistosomiasis-attributable benefit of treatment for anemia difficult, though most authors suggest that a portion of observed improvement in hemoglobin was likely due to reduced schistosome burden.

Only two randomized controlled trials have been conducted utilizing monotherapy with a drug efficacious predominantly against schistosomes [68, 69]. One study conducted in an *S. japonicum* endemic village in Leyte, the Philippines demonstrated significant differences in hemoglobin six months after therapy between children in the intervention and control arms. The magnitude of effect was approximately 1.1 g/dL. Of note, males experienced a greater increase in hemoglobin with treatment than did females. The experimental design of this study and the “isolation” of schistosomiasis support a role for *S. japonicum* in the etiology of anemia in regions of the developing world where it is endemic. Another randomized controlled trial examined praziquantel treatment alone for *S*. *haematobium*, and found no effect of treatment on hemoglobin [68]. This is unlikely to be due to lack of statistical power (*N* = 104 in placebo and 105 in praziquantel), low prevalence of anemia (70%), or inadequate followup (eight months). However, it is possible that this null finding was due to the exclusion of children with high intensity infections.

### 4.1. Anemia of Inflammation

Anemia of inflammation must be mentioned in the context of iron status in LDCs, as this process leads to both decreased iron absorption and sequestration of iron into storage forms, rendering it less available to host tissues. Further, the etiology of anemia (iron deficiency anemia or anemia of inflammation, or both) is difficult to determine in the developing world context where assays to determine biomarkers of iron status are not available. Anemia of inflammation is characterized by decreased red blood cell production through a series of mechanisms, mediated in part by proinflammatory cytokines, particularly TNF-alpha and IL-6. These mechanisms include suppression of the normal response of bone marrow to erythropoietin, decreased synthesis of erythropoietin, dyserythropoiesis (disturbances of bone marrow cellular division), and alterations in iron metabolism such that iron is sequestered into storage forms, such as ferritin, making it less bio-available [70]. 

Recently, major advances have been made in our understanding of anemia of inflammation [71]. In response to inflammation, in particular IL-6, hepcidin is synthesized [72]. This protein has pleiotropic effects on iron metabolism. Hepcidin causes sequestration of iron from bio-available forms to storage forms, as well as decreased intestinal absorption of iron [73, 74]. Both processes are due to the binding of hepcidin to ferroportin, the only iron egress protein in humans. Hepcidin-ferroportin binding causes the internalization and degradation of ferroportin [75], thus trapping iron inside cells. This results in the inability to absorb iron from the gut and to mobilize iron from reticuloendothelial macrophages [73, 76, 77]. In the proximal small intestine, iron is trapped in intestinal epithelial cells and lost during normal cell turnover [74, 78]. Decreased absorption and iron sequestration leads to decreased iron bio-availability to meet needs such as erythropoeisis. This is of great public health importance given that anemia of inflammation contributes to, or is the primary cause of, anemia in the context of many common infectious diseases in LDCs including malaria, HIV, schistosomiasis, tuberculosis, and others [7, 14, 62, 79, 80].

### 4.2. HIV

While HIV is not a significant cause of iron deficiency anemia, its role as a major cause of anemia in LDCs warrants special mention due to the “functional” iron deficiency caused by HIV-related anemia of inflammation. Anemia is highly prevalent among people with HIV-infection, and has been identified as a marker for disease progression and associated with decreased survival [3, 81–83]. There is significant evidence to support anemia of inflammation as the primary cause of anemia in the context of HIV infection [3]. A study conducted among infants in Uganda found the prevalence of IDA (Hb < 11.0 g/dL and ferritin < 12 g/L) to be approximately equal among HIV-infected and uninfected infants, with prevalence of any anemia significantly higher in the HIV-infected group than the uninfected group (90.9% and 76.9%, resp.). The authors attribute the excess anemia among HIV-infected infants to anemia of inflammation [3, 84]. Another study conducted among antiretroviral-naïve HIV-infected children in South Africa found that prevalence of anemia increased with HIV disease stage from 42% for children with no evidence of immunosuppression to 85% for children with severe immunosuppression. Iron deficiency anemia was present in only 17% of the children, further supporting the primary role of anemia of inflammation in the pathogenesis anemia in the context of HIV infection [3, 85].

## 5. Trichuris

The prevalence of infection with *T. trichiura *in the developing world is high, approaching 95% in areas of endemnicity and affecting an estimated 1049 million people [86]. Although low-intensity infections with *T. trichiura* appear to be asymptomatic, high-intensity infections and Trichuris Dysentery Syndrome (TDS) are associated with growth stunting and anemia [87, 88]. Potential mechanisms of trichuris-related anemia include worm consumption of blood in the context of heavy infections, colonic lesions with associated bleeding, or chronic reduction in food and micronutrient intake due to the anorexia inducing effects of TNF-alpha released in response to infection [86, 89]. Of note, one study assessing occult blood loss with *T. trichiura *found that trichuriasis does not lead to significant occult gastrointestinal bleeding in the absence of TDS [90].

## 6. Bacterial Dysentery Syndromes

Occult blood loss due to Shigellosis or Enteroinvasive *Escherichia coli * dysentery may contribute to iron deficiency anemia among infants and children in areas of the developing world where these pathogens cause frequent childhood illness. Absolute blood loss due to dysentery or its contribution to IDA have not been well quantified.

## 7. *Helicobacter pylori*

The greatest prevalence of *H. pylori *is in LDCs, where the majority of infections are acquired during early childhood [91, 92]. *H. pylori *neutralizes and decreases secretion of gastric acid resulting in hypochlorhydria, and causes chronic inflammation of the gastrointestinal tract. Studies conducted among both adults and children have implicated *H. pylori *as a cause of iron deficiency anemia [92–97]. It is hypothesized that *H. pylori*-associated iron deficiency anemia is caused by both compromised absorption of bioavailable iron in the context of hypochlorhydria, and the competing iron demands of *H. pylori *and the host [92, 98, 99].

## 8. Malaria

Malaria is a major contributor to anemia in the developing world. Though the primary cause of anemia in the context of malaria is hemolytic, studies have demonstrated that anemia of inflammation plays an important role as well by inducing changes in iron absorption and distribution [14, 100]. Both clinical and asymptomatic malaria have been associated with anemia of inflammation. In a study in Indonesia, de Mast et al. found that asymptomatic children with *P. falciparum *or *P. vivax *parasitemia had significantly lower hemoglobin concentrations than aparasitemic controls (12.6 and 12.2 g/dL versus 14.4 g/dL; *P* < .01), as well as significantly higher serum hepcidin concentrations (5.2 and 5.6 nM versus 3.1 nM; *P* < .05) [100]. These results support the concept that prolonged sequestration of iron into storage forms due to inflammation caused by malaria parasitemia is likely a major cause of anemia in the developing world. 

Exceptionally high rates of maternal morbidity and mortality are a major concern in the developing world, particularly in sub-Saharan Africa [101]. Much of this excess morbidity and mortality is attributable to malaria [102, 103]. Risk of malarial infection is especially high in HIV-infected individuals [104]. Studies have shown that HIV infection contributes to the burden of malarial anemia by increasing risk for parasitemia, severe anemia, and treatment failure [3]. Women are more susceptible to *P*. *falciparum* malaria during pregnancy, and the risk of disease and death is high for both the mother and her fetus. In areas of stable malaria transmission, an estimated one in four women has evidence of malarial infection at the time of delivery [105]. This underestimates the burden throughout gestation as it is ascertained only at delivery and does not take into account effects of infections preceding delivery [103]. In stable transmission areas, severe maternal anemia and low birth weight are frequent sequelae and account for an enormous loss of life [106, 107]. To address this, WHO recommends provision of intermittent prophylactic treatment in pregnancy (IPTp) to reduce the risk of pregnancy malaria and related in malaria-endemic regions. IPTp entails provision of sulfadoxine-pyrimethamine (SP) during the second and third trimester [107]. Early studies found that SP-IPTp significantly reduced the frequency of both placental and peripheral parasitemia [108–110], increased maternal hemoglobin levels, and reduced the prevalence of moderate to severe anemia [108, 111]. Approximately one in four cases of severe maternal anemia may be prevented with adequate prevention of malaria during pregnancy [112]. There is growing concern, however, with respect to malaria resistance to SP and the efficacy of this regimen [113]. New studies are underway to evaluate the safety and efficacy of newer antimalarial drugs in pregnancy. 

Treatment for malaria and anemia in LDCs is further complicated by concerns that provision of iron in malaria endemic regions may increase the risk for malaria-related morbidity and mortality [114–116]. Studies in Tanzania found that pregnant women who were iron-replete versus iron-deficient at delivery were three times more likely to have placental parasitemia [114, 117]. In addition, among iron-replete mothers, placental malaria led to an increased risk of anemia among newborns [117]. The deleterious interaction between iron and malaria has also been demonstrated among children. Iron plus folate supplementation given during a recent randomized, placebo-controlled trial in a high *P. falciparum* transmission area of Zanzibar resulted in a 15% increase in all cause mortality [116]. Enhanced malarial morbidity was concentrated in children who were iron-replete at baseline. Children receiving iron and folate suffered 16% (*P* = .02) greater risk of clinical malaria and a 70% greater risk (*P* = .02) of cerebral malaria. In a separate study in Kenya, clinical malaria was significantly less frequent among iron-deficient children [118]. In Papua New Guinea, iron supplementation increased the prevalence of parasitemia and splenomegaly among infants [119]. Iron supplementation did not, however, increase the risk of malaria among children under intensive health surveillance in Kenya [120]. Taken together, these findings suggest that routine supplementation with iron may place populations in malaria endemic settings at increased risk for malaria-specific morbidity and mortality [116]. Effects of baseline iron status, acquired immunity, or intensity of health surveillance could explain some of the variability in results. Further studies are warranted and underway to more clearly evaluate the balance of risks and benefits and potential effect modifiers of this risk for specific populations.

## 9. Iron Supplementation in Pregnancy

Despite WHO recommendations to provide prenatal vitamins with iron [121], studies do not support an association between iron interventions and birth outcomes [122, 123]. A recent Cochrane review of this topic reaffirmed this conclusion [124], and a separate earlier review concluded that “the currently available evidence from studies with designs appropriate to establish a causal relationship is insufficient to support or reject the practice (prenatal iron supplementation) for the specific purposes of raising birth weight or lowering the rate of preterm birth” [122]. This is further supported by animal data including a primate study of iron supplementation during pregnancy [125]. In that study using Rhesus monkeys, neonatal neurobehavioral test scores, number of stillbirths, birth weight, and maternal weight gain were not significantly different between dams fed a diet containing iron (100 *μ*g Fe/g) and those monkeys deprived of iron (<10 *μ*g Fe/g). Further, maternal monkeys receiving the iron-replete diet did differ significantly in hematocrit, MCV, transferrin saturation, serum ferritin, and serum iron compared to those receiving an iron-deplete diet. However, newborn monkeys did have higher hemoglobin values with maternal supplementation. 

Ongoing discrepancies across studies with respect to benefits of iron supplementation during pregnancy are likely due to the underrecognized role of anemia of inflammation in the developing world. This is highlighted by a recent study that examined a wide range of nutritional and nonnutritional causes of anemia in pregnancy in Malawi, including bone marrow aspiration [6]. Investigators found that CRP concentrations were elevated in 54% of anemic pregnant women with *no nutritional deficiencies* and in 73.5% of the anemic women who were iron-replete by bone marrow assessment. This study demonstrates the important role played by anemia of inflammation in the pathogenesis of anemia in pregnancy. In the context of anemia of inflammation, the absorption of prenatal iron supplements will be limited and absorbed iron will be shunted from bio-available forms to storage forms, limiting maternal use and potentially inhibiting delivery of iron to the fetus. The possible exacerbation of malaria-related morbidity in pregnancy associated with provision of prenatal iron may also explain disparate findings, if iron provision in this context is increasing malarial anemia [114, 117]. 

Other benefits of prenatal iron supplementation have been identified, however. In particular, many studies have demonstrated improvements in maternal hemoglobin with prenatal iron supplementation [123]. This may be of greater significance in the developing world where there is a higher prevalence of moderate to severe iron deficiency anemia with consequent greater risk of perinatal mortality related to postpartum hemorrhage. Prenatal iron supplementation may also have beneficial effects for the newborn. Preziozi et al. found that newborn iron status is dependent on maternal iron status during pregnancy and that children born to iron-treated mothers have higher serum ferritin, greater body length, and increased Apgar scores in comparison to those treated with placebo [126]. Other studies conducted in LDCs have shown that concentrations of hemoglobin, iron, and ferritin are significantly lower in the cord blood newborns of anemic mothers, with linear relationships demonstrated with maternal hemoglobin and ferritin levels [2]. 

### 9.1. Diagnosis and Treatment

Despite the enormous global burden of disease due to anemia and the multiple causes of anemia in LDCs, diagnostic tests to distinguish primary etiologies are limited, particulary in these cost-constrained settings. Given iron deficiency anemia (due to dietary insufficiency and infectious diseases) and anemia of inflammation are the two leading causes of anemia in LDCs, and interventions to address these differ, diagnostic tools to differentiate the two are of paramount importance. 

Until recently, there has been a lack of valid biomarkers to distinguish the causes of anemia. Hemoglobin and ferritin have proven to be poor diagnostic tools [127], largely due to the fact that ferritin is decreased in the context of iron deficiency, but increased in the context of anemia of inflammation as iron is sequestered in storage forms. The emergence of hepcidin as a biomarker for anemia of inflammation may help to fill this diagnostic gap, and assist in effectively selecting individuals who will benefit most from iron therapy [100]. Treatment of anemia of inflammation requires diagnosis and treatment of the underlying causes of inflammation, highlighting the importance of treatment programs for HIV, helminth infections and malaria (both clinical and subclinical) in curbing anemia in the LDCs.
